# 1,5-Diazacyclohenicosane, a New Cytotoxic Metabolite from the Marine Sponge *Mycale* sp

**DOI:** 10.3390/md7030445

**Published:** 2009-09-15

**Authors:** Laura Coello, María Jesús Martín, Fernando Reyes

**Affiliations:** Medicinal Chemistry Department, PharmaMar S.A.U., Pol. Ind. La Mina Norte, Avda de los Reyes 1, 28770-Colmenar Viejo (Madrid). Spain; E-Mails:lcoello@pharmamar.com (L.C.);mjmartin@pharmamar.com (M.J.M.)

**Keywords:** marine sponges, alkaloids, cytotoxicity, Mycale sp

## Abstract

A new cyclic diamine, 1,5-diazacyclohenicosane (**1**), was isolated from samples of the marine sponge *Mycale* sp. collected at Lamu Island (Kenya). Its structure was determined by a combination of spectroscopic techniques, including (+)-HRESIMS and 1D and 2D NMR spectroscopy. The compound displayed cytotoxicity at the μM level against three human tumor cell lines.

## 1. Introduction

Sponges continue to be a rich source of metabolites with interesting biological properties [1,2]. Among them, specimens belonging to the genus *Mycale* have been the subject of extensive research leading to the isolation and identification of a wide variety of novel structures. Some of these *Mycale* metabolites display interesting biological properties such as the antiviral and antitumour activities detected in the mycalamides [3,4], the potent cytotoxicity exhibited by the mycalolides [5–7], pateamine [8], and peloruside A [9] or the histone deacetylase inhibitory properties displayed by the cyclic tetrapeptides azumamides A–E [10].

As part of our continuing program to search for new anticancer agents, the chemical composition of samples of the marine sponge *Mycale* sp. collected at Lamu Island (Kenya) was investigated due to the cytotoxicity displayed by their organic extracts. Herein we report the isolation, structural characterization and cytotoxic activity of a new cyclic diamine, 1,5-diazacyclohenicosane (**1**), obtained by bioassay-guided fractionation of extracts of this sponge.

## 2. Results and Discussion

**Figure 1:**
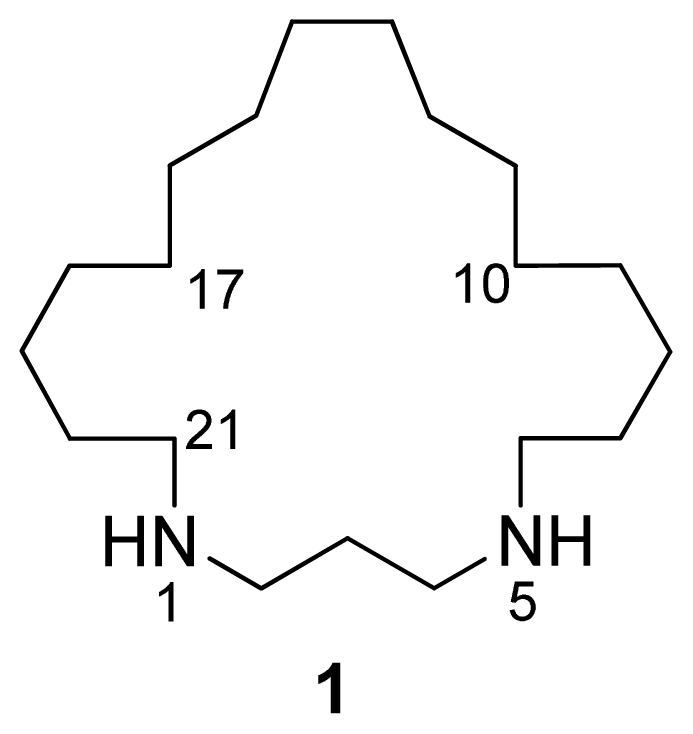
Structure of compound **1**.

Freshly collected samples of *Mycale* sp. were immediately frozen and transported stored in dry ice to PharmaMar. The frozen sponge was triturated and extracted with a 1:1 mixture of MeOH:CH_2_Cl_2_, and the organic extract subjected to reversed-phase VLC on Polygoprep C18 silica gel to yield a bioactive fraction containing compound **1** (Figure 1).

**Figure 2:**
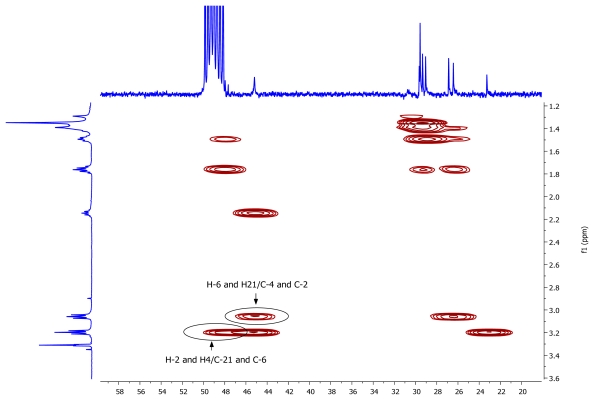
Key HMBC correlations for compound **1**.

A pseudomolecular ion at *m/z* 297.3266 in the (+)-HRESIMS of **1** accounted for a molecular formula of C_19_H_40_N_2_ (calcd. for C_19_H_41_N_2_ 297.3264), requiring one degree of unsaturation. The existence of an element of symmetry in the structure of the compound was evidenced by the presence of only nine resonance signals, one of them with double intensity, in its ^13^C-NMR spectrum (Table 1). Only methylene groups were present in the structure of **1**, according to the edited HSQC spectrum. The chemical shifts of two of the ^13^C signals (48.2 and 45.2 ppm), accounting for four carbon atoms, indicated that these atoms were attached to nitrogen. COSY correlations measured in CD_3_OD (Table 1) established the presence of two spin systems in the molecule: from C-2 to C-4 and from C-6 to C-21. Finally, key HMBC cross-peaks observed between H-2/H-4 and C-21/C-6, and between H-6/H-21 and C-4/C-2 (Figure 2) connected both spin systems and established the identity of compound **1** as 1,5-diazacyclohenicosane.

The cytotoxic activity of **1** was tested against three human tumor cell lines, including lung (A549), colon (HT29) and breast (MDA-MB-231) tissues. The compound exhibited moderate activity with GI_50_ values in the micromolar range and no selectivity against the cell lines tested: 5.41 μM (A549), 5.07 μM (HT29) and 5.74 μM (MDA-MB-231). Doxorubicin displayed values of 0.32 μM (A549), 0.36 μM (HT29) and 0.26 μM (MDA-MB-231) when tested as a positive control under the same conditions.

In summary, a new cytotoxic cyclic diamine, 1,5-diazacyclohenicosane (**1**), has been isolated from samples of the Kenyan sponge *Mycale* sp. Its structure resembles that of other cyclic amines previously characterized from sponge samples such as the motuporamines isolated from the Papua New Guinea sponge *Xestospongia exigua* [11–12] or halichlorensin obtained from South African specimens of *Halichlona tulearensis* [13–14]. This structural similarity together with the interesting anti-invasive and anti-angiogenic properties displayed by the motuporamine family suggest that further studies to assess the potential of our compound in this area of research are merited.

## 3. Experimental Section

### 3.1. General

NMR spectra were recorded on Varian “Unity 500” (500 MHz, ^1^H) or Varian “Unity 300” (75 MHz, ^13^C) spectrometers. Chemical shifts were reported in ppm using residual CD_3_OD (δ 3.31 for ^1^H and 49.0 for ^13^C) as internal reference. HMBC experiments were optimized for a ^3^*J*_CH_ of 8 Hz. (+)-HRESIMS was performed on a QSTAR Applied Biosystems spectrometer. (+)-ESIMS were recorded using an Agilent 1100 Series LC/MSD spectrometer.

### 3.2. Animal material

*Mycale* sp. was collected in August 2005 by SCUBA diving at depths between 8 and 24 m at Lamu Island (Kenya) (3º 37′ 07″ S, 39º 53′ 43″ E). The material was identified by Dr. José Luis Carballo from the Universidad Nacional Autónoma (México). A voucher specimen is deposited at PharmaMar (ORMA037245).

### 3.3. Extraction and isolation

The frozen organism (14 g) was triturated and extracted with a 1:1 mixture of CH_2_Cl_2_:MeOH (3 × 200 mL). The extract was concentrated to yield a crude of 1.20 g. This material was subjected to VLC on Polygoprep RP-18 with a stepped gradient from H_2_O:MeOH 3:1 to MeOH. The bioactive fraction eluted with H_2_O:MeOH 1:3 contained pure compound **1** (12.9 mg).

*1,5-Diazacyclohenicosane* (**1**). Pale yellow amorphous solid; ^1^H- (500 MHz) and ^13^C-NMR (75 MHz) see Table 1; (+)-ESIMS *m/z* 297 [M+H]^+^; (+)-HRESIMS m/z 297.3266 [M+H]^+^ (calcd. for C_19_H_41_N_2_, 297.3264).

### 3.4. Biological activity

A549 (ATCC CCL-185), lung carcinoma; HT29 (ATCC HTB-38), colorectal carcinoma and MDA-MB-231 (ATCC HTB-26), breast adenocarcinoma cell lines were obtained from the ATCC. Cell lines were maintained in RPMI medium supplemented with 10% fetal calf serum (FCS), 2 mM l-glutamine and 100 U/mL penicillin and streptomycin, at 37 ºC and 5% CO_2_. Triplicate cultures were incubated for 72 h in the presence or absence of test compounds (at ten concentrations ranging from 10 to 0.0026 μg/mL). For quantitative estimation of cytotoxicity, the colorimetric sulforhodamine B (SRB) method was used [15]. Briefly, cells were washed twice with PBS, fixed for 15 min in 1% glutaraldehyde solution, rinsed twice in PBS, and stained in 0.4% SRB solution for 30 min at room temperature. Cells were then rinsed several times with 1% acetic acid solution and air-dried. Sulforhodamine B was then extracted in 10 mM trizma base solution and the absorbance measured at 490 nm. Results are expressed as GI_50_, the concentration that causes 50% inhibition in cell growth after correction for cell count at the start of the experiment (NCI algorithm). Doxorubicin and DMSO (solvent) were used as the positive and negative controls in this assay. Prism 3.03 from GraphPad was used for the statistical analysis of the cell growth inhibition results.

## Figures and Tables

**Table 1:** NMR data of compound **1** (CD_3_OD, 500/75 MHz).

Nº	δ_C_, mult.	δ_H_, mult. (*J* in Hz)	COSY	HMBC
2	45.2, CH_2_	3.20, t (7.2)	3	3, 4, 21
3	23.3, CH_2_	2.15, quint (7.2)	2, 4	2, 4
4	45.2, CH_2_	3.20, t (7.2)	3	2, 3, 6
6	48.2, CH_2_	3.06, m	7	4, 7, 8
7	26.4, CH_2_	1.76, m	6, 8	6, 8, 9
8	26.9, CH_2_	1.49, m	7, 9	6,7, 9, 10
9	29.3, CH_2_	1.39, m		
10	29.1, CH_2_	1.35, m		
11	29.6, CH_2_*	1.35, m		
12	29.6, CH_2_*	1.35, m		
13	29.7, CH_2_*	1.35, m		
14	29.7, CH_2_*	1.35, m		
15	29.6, CH_2_*	1.35, m		
16	29.6, CH_2_*	1.35, m		
17	29.1, CH_2_	1.35, m		
18	29.3, CH_2_	1.39, m		
19	26.9, CH_2_	1.49, m	18, 20	17, 18, 20, 21
20	26.4, CH_2_	1.76, m	19, 21	18, 19, 21
21	48.2, CH_2_	3.06, m	20	2, 19, 20

* Interchangeable assignments.
